# A model based on age, sex, and morbidity to explain variation in UK general practice prescribing: cohort study

**DOI:** 10.1136/bmj.a238

**Published:** 2008-07-14

**Authors:** Rumana Z Omar, Caoimhe O’Sullivan, Irene Petersen, Amir Islam, Azeem Majeed

**Affiliations:** 1 Department of Statistical Science, University College London, London WC1E 6BT; 2 University College London Hospital/University College London Biomedical Research Unit, University College London Hospitals NHS Trust, London W1P 9LL; 3 Department of Primary Care and Population Sciences, University College London; 4 Department of Primary Care and Social Medicine, Imperial College London

## Abstract

**Objective** To examine whether patient level morbidity based measure of clinical case mix explains variations in prescribing in general practice.

**Design** Retrospective study of a cohort of patients followed for one year.

**Setting** UK General Practice Research Database.

**Participants** 129 general practices, with a total list size of 1 032 072.

**Main outcome measures** Each patient was assigned a morbidity group on the bases of diagnoses, age, and sex using the Johns Hopkins adjusted clinical group case mix system. Multilevel regression models were used to explain variability in prescribing, with age, sex, and morbidity as predictors.

**Results** The median number of prescriptions issued annually to a patient is 2 (90% range 0 to 18). The number of prescriptions issued to a patient increases with age and morbidity. Age and sex explained only 10% of the total variation in prescribing compared with 80% after including morbidity. When variation in prescribing was split between practices and within practices, most of the variation was at the practice level. Morbidity explained both variations well.

**Conclusions** Inclusion of a diagnosis based patient morbidity measure in prescribing models can explain a large amount of variability, both between practices and within practices. The use of patient based case mix systems may prove useful in allocation of budgets and therefore should be investigated further when examining prescribing patterns in general practices in the UK, particularly for specific therapeutic areas.

## Introduction

The prescribing costs of general practitioners in the United Kingdom have increased rapidly in recent years, with a 60% real terms increase in spending since 1996 and a 55% increase in the number of items dispensed. Prescribing by general practitioners now costs around £7.8b (€9.9; $15.3) a year, about 10% of the National Health Service’s expenditure in England.^1^ General practitioners’ prescribing decisions are coming under increasing scrutiny, with considerable pressure to prescribe cost effectively.^2^ The development of new drugs, enhanced indications for existing drugs (such as statins), more rigorous management of chronic diseases, and the ageing of the population of England will all continue to increase the cost and volume of prescribing in primary care.^3^ Prescribing budgets for primary care trusts are now allocated using a needs based formula. Budgetary allocations for prescribing to general practices are, however, still largely based on historical prescribing patterns.^4^ When these patterns do not reflect clinical need, historical inequities in resource allocations are perpetuated.

To overcome these problems some primary care trusts are now using needs based models to determine indicative prescribing budgets for general practices. A limitation of these models is that they are largely based on the demographic profile of a practice population, sometimes with a weighting for local characteristics taken from the census. The models do not generally contain any direct measure of morbidity within a practice. Previous research on these models has generally shown that they are poor predictors of prescribing costs in practices; and general practices with high prescribing costs often come under considerable pressure to reduce these costs.^5^ Consequently, general practices that look after populations with higher burdens of morbidity may be unfairly scrutinised or penalised for having high prescribing rates. Variation in prescribing could be due to differences in the case mix of patients registered with the general practices, socioeconomic factors, or inefficient or inappropriate prescribing. More sophisticated models to explain these variations are needed. Prescribing models that incorporate morbidity could be used to help predict expenditure for budgetary planning and separate practices that have high prescribing costs because of a high burden of disease from those that have high costs because of inefficient prescribing. These models could also help identify practices that are under-treating patients and that have inappropriately low prescribing rates for their practice’s morbidity burden.

We used the Johns Hopkins adjusted clinical group case mix system^6^ to investigate how well patient level morbidity based measures of case mix explain the variability in prescribing among general practices in the UK. This is the only case mix system specifically designed for use in primary care and it has been widely used in studies examining variations in primary care practice.^7^ ^8^ ^9^ ^10^

## Methods

We obtained data from the UK General Practice Research Database.^11^ General practices participating in the database follow set guidelines for the recording of clinical and prescribing data and submit anonymised patient based clinical records to the database at regular intervals. The accuracy and comprehensiveness of the data recorded in the database has been documented previously.^12^ ^13^ The variables collected by the database include age; sex; registration details; medical diagnoses (Read and OXMIS codes) that are part of routine care or resulting from admissions to hospital, consultations or emergency care; referrals; laboratory tests; and prescriptions issued for each patient. Although the prescriptions issued by specialists are not picked up in the General Practice Research Database, most prescriptions for chronic disease in the UK are issued by general practitioners. Data for the year 2001 were obtained only for practices that met the “up to standard criteria,” a quality marker set on the basis of internal consistency of the practice, completeness of longitudinal recording, and compliance with the recording guidelines of the General Practice Research Database.^14^ All practices provided data for the entire one year period. We excluded patients if they were registered with a practice for less than 180 days. Using a lookup table we converted the Read and OXMIS codes to those of the *International Classification of Diseases,* ninth revision.^15^ ^16^

To construct the morbidity groups we used the Johns Hopkins adjusted clinical group system software^5^ to initially assign the patients into one of the 81 mutually exclusive Johns Hopkins adjusted clinical groups, on the basis of age, sex, and a combination of recorded diagnoses over a one year period. We then assembled these groups into six mutually exclusive categories using the range of diagnoses pertaining to each patient. These six categories are constructed by the software according to patients’ expected resource use on the basis of a nationally representative database of 2 million patients aged less than 65 years in the United States. For example, a patient with uncomplicated type 2 diabetes would be placed in group 2, whereas a patient with type 2 diabetes, heart failure, cellulitis, and chest pain would be placed in group 5.^6^ In this paper we used these six groups to represent patient morbidity groups, with group 1 being the healthiest patients and group 6 the sickest. Age was grouped as children (0-15 years), young adults (16-34), older adults (35-64), and adults of pensionable age (≥65 years).

Using the rule of 10 events or observations required per coefficient estimated in a model and adjusting for the design factor (using intracluster correlation coefficient of 0.02 for prescribing and average cluster size of 8000), our study required a total of 14 000 events or observations to estimate the models’ coefficients with adequate precision.^17^ After exclusions, the dataset used from the General Practice Research Database had more than sufficient numbers of events or observations.

### Statistical analysis

We used a two level Poisson model with random intercepts to investigate the association between age, sex, morbidity, and the number of prescriptions issued^18^ (outcome and predictors were considered at the patient level), after accounting for clustering within the general practices.

Initially we estimated the extent of variation in prescribing at the practice level that is explained by the predictors, using an adjusted R^2^ measure based on a linear regression model.^19^ This was a practice level analysis in which we considered as the outcome the mean number of prescriptions issued by each practice and used the practice mean for age and proportions for each sex and morbidity groups as predictors. To partition the variation in prescribing at practice and patient levels we used an R^2^ measure derived from a two level logistic regression with random intercepts.^20^ For this purpose we converted the number of prescriptions to a dichotomous response according to whether or not a patient had received a prescription. As some information may be lost owing to collapsing number of prescriptions to categories or mean, we carried out a sensitivity analysis to check the consistency of the results using another type of R^2^ measure estimated from a two level linear regression model with random intercepts.^20^ We used a square root transformation of the number of prescriptions issued as the response to satisfy the assumptions of normality. We compared the R^2^ measures obtained from all three methods across models fitted with no predictors, with age and sex, and with age, sex, and morbidity.

To assess how well the models discriminated between patients who had received a prescription and those who had not we calculated the receiver operating characteristic areas from the logistic model.^21^ The receiver operating characteristic area represents the proportion of patient pairs that is correctly ranked by a model according to the prescribing status of the patients.

We used residual plots to investigate assumptions of normality of residuals required by the multilevel models. MlwiN v2.02 software^22^ and Stata version 9.2^23^ were used for the statistical analyses.

## Results

Information on age, diagnosis, and prescribing was complete. Twelve patients were of indeterminate sex and were excluded from the analysis. After exclusions, 129 practices with 1 032 072 patients were eligible for inclusion. The median time that a patient had been registered with a general practitioner in 2001 was 11 years. Overall, 49.3% of the patients were male and 50.7% were female. Sixty four per cent of patients were issued a prescription at least once during 2001. The median percentage of patients issued a prescription by the practices was 65% (90% range 11% to 75%). The median number of prescriptions issued to a patient across the 129 practices was 2 (0 to 18). The median total number of prescriptions issued across the 129 practices was 9852 (3508 to 14 589).

The number of patients in the two sickest morbidity groups was small and therefore combined in all analyses. The results from table 1 show the variations in the age, sex, and morbidity distribution of patients across practices along with the number of prescriptions issued across all practices for each of these groups. The sex distribution of the patients was similar across the practices. The age and morbidity distributions of patients varied, however, particularly for those in the oldest age group (≥65 years) and for morbidity groups 4-6. The median number of prescriptions issued increased with age and morbidity groups and was higher for females. The number of prescriptions issued by the practices varied considerably, with the highest variation occurring in patients aged more than 65 and in the sickest morbidity groups.

**Table 1 tbl1:** Number of patients and annual number of prescriptions issued by age, sex, and morbidity

Variable	No of patients	Median %* (90% range) across practices	Annual No of prescriptions	Median of annual No of prescriptions (90% range) across practices
Age group (years):				
0-15	202 303	19.0 (15.3 to 25.6)	392 437	1 (0 to 8)
16-34	257 806	24.8 (18.4 to 35.0)	624 181	1 (0 to 10)
35-64	407 051	39.5 (32.7 to 43.7)	1 768 563	2 (1 to 17)
≥65	164 912	15.9 (8.2 to 22.2)	1 840 789	10 (0 to 28)
Sex:				
Male	508 545	49.3 (47.4 to 52.3)	1 831 839	1 (0 to 17)
Female	523 527	50.7 (47.7 to 52.6)	2 794 131	3 (0 to 19)
Morbidity:				
1 (healthiest)	338 890	31.1 (23.9 to 46.0)	24 648	0
2	140 972	13.7 (8.7 to 20.5)	483 762	2 (0 to 13)
3	251 278	25.0 (20.2 to 28.1)	1 177 099	3 (0 to 15)
4	274 814	27.1 (13.6 to 35.0)	2 602 883	7 (1 to 25)
5 and 6 (sickest)	26 118	2.5 (1.1 to 4.5)	337 578	9 (1 to 36)
Overall	1 032 072		4 625 970	2 (0 to 18)

*Percentage of patients in each age, sex, and morbidity groups were calculated for each practice.

The number of prescriptions issued to a patient was strongly associated with the patient’s age and morbidity (table 2; P<0.001), increasing steeply with age and morbidity. Several scenarios are used to illustrate the relations observed in these models. The expected number of prescriptions for boys and girls aged 0 to 15 are estimated to be 1.6 and 2.2, respectively, whereas the expected numbers for men and women aged 65 or more are 9.2 and 12.7. For the healthiest boys and girls aged 0 to 15 the expected number of prescriptions is 0.05 (same for both). The corresponding values for the least healthy girls and boys are 6.2 and 6.8. The expected numbers of prescriptions for the least healthy men and women aged more than 65 are 21.1 and 23.3.

**Table 2 tbl2:** Association between age, sex, and morbidity and number of prescriptions issued (results from two level Poisson regression models using patient level data)

Variable	Rate ratio (95% CI)	
	Model 2*	Model 3†
Age group (years):		
0-15	1	1
16-34	1.26 (1.25 to 1.26)	1.13 (1.12 to 1.13)
35-64	2.26 (2.25 to 2.27)	1.85 (1.84 to 1.86)
≥65	5.65 (5.63 to 5.67)	3.38 (3.37 to 3.39)
Sex:		
Male	1	1
Female	1.38 (1.37 to 1.38)	1.10 (1.10 to 1.11)
Morbidity:		
1 (healthiest)	—	1
2	—	43.42 (42.83 to 44.02)
3	—	58.21 (57.53 to 58.89)
4	—	97.03 (95.89 to 98.18)
5 and 6 (sickest)	—	134.56 (132.73 to 136.42)

Number of prescriptions issued for each patient was considered as response variable.

*Age and sex.

†Age, sex, and morbidity.

Table 3 presents the results on the extent of variation explained in prescribing from the practice level analysis. Adding morbidity explains more of the variation in prescribing between practices. This result is supported by the patient level analysis presented in table 4 where variation is split into practice and patient levels. The inclusion of morbidity explained considerably more of the total variability than patients’ age and sex alone (80% *v* 10%). Of the total variation, only 0.1% remained unexplained at the practice level and 19% remained unexplained at the patient level, after adjusting for age, sex, and morbidity. When adjusting for only age and sex the corresponding values are 4% and 86%. The results also show that most (96%) of the total variation was within practices. The extent of variation explained in prescribing based on the sensitivity analysis was 60% at patient level and 74% at practice level when morbidity was included and 20% and 6% when only age and sex were included.

**Table 3 tbl3:** Percentage of variation between practices in prescribing explained using practice level measures

Regression models	Variation (%) explained at practice level
Model 1: no predictors	0
Model 2: age and sex	4
Model 3: age, sex, and morbidity	57

Mean number of prescriptions issued by each practice was used as response. Predictors were summarised to express mean (for age) and percentage (for sex and morbidity) for each practice.

**Table 4 tbl4:** Percentage of variation in prescribing explained using patient level data

Variation	Model 1* (%)	Model 2† (%)	Model 3‡ (%)
Percentage of total variance explained	0	9.7	80.1
Level at which % of total variance was unexplained:			
Practice level	3.9	4.1	0.1
Patient level	96.1	86.2	19.0

Prescribing was dichotomised as prescription issued or not issued for each patient.

*No predictors.

†Age and sex.

‡Age, sex, and morbidity group.

The receiver operating characteristic area for a model with age and sex was 0.648 (95% confidence interval 0.647 to 0.649), which increased to 0.972 (0.971 to 0.972) when morbidity was included. Thus morbidity significantly improved the ability of the model to discriminate between patients who had received prescriptions and those who had not.

## Discussion

Patient morbidity explains considerably more of the variability in prescribing than patients’ age and sex alone. About 4% of the total variation is at the practice level and most of the variation is within practices.

### Comparison with previous studies

Studies have shown that prescribing in general practice varies considerably, with threefold to fourfold variations commonly seen even after practices with outlying prescribing rates are excluded. Statistical models from these studies have not included direct measures of case mix and have generally explained only a small proportion of this variation. Other than morbidity within a practice other factors that could influence prescribing rates include deprivation; doctors’ knowledge, professional experience, role perception, and time pressures; the number of doctors in the general practice; and patients’ expectations of receiving a prescription and their demands.^24^ ^25^ ^26^ ^27^ ^28^ ^29^

### Strengths and limitations

We used data from the General Practice Research Database, which has been extensively validated and shown to be of high quality. The practices submitting information to the database are reasonably representative of the age and sex profile of the UK population, with some under-representation of inner city practices. The average size of the practices is greater than the national average.^13^ ^30^ In contrast with many previous studies of variation in prescribing, this study used data at individual patient level rather than an ecological design. The ecological design has the limitation of drawing inferences at the individual patient level solely on the basis of aggregate statistics. This study also controlled for diagnosis based morbidity groupings specifically designed for use in primary care when examining variation in prescribing.

Among the limitations of the study is that the adjusted clinical group system was developed for use in the United States and therefore might need some further adaptation to maximise its utility in the UK. It has, however, now been used for an increasing number of UK based studies. Finally, the adjusted clinical group system depends on diagnostic codes recorded by the general practitioners during consultations. Differences in the way that general practitioners record similar conditions on their practice computers could introduce bias into the estimates of their practices’ morbidity scores.

### Implications for practice

This study used patients’ clinical case mix to explain variation in general practice prescribing. Including morbidity in the model considerably improves its explanatory power and therefore its potential utility for monitoring prescribing in general practice and the allocation of prescribing budgets. With the increasing use of electronic medical records in general practice, computerised clinical data for activities such as measurement of case mix and assessment of morbidity will become increasingly available.

This study shows how well morbidity helped in explaining variation in the number of prescriptions issued and in determining which group of patients are most likely to receive prescriptions. Each prescription issued might, however, contain several items and contain drugs for very different therapeutic areas. Hence further work is required to investigate the association between morbidity and total prescribing volume (measured by the number of items prescribed) and costs and how well morbidity explains variation in prescribing in specific therapeutic areas. The use of such patient based measures of case mix could then be explored in setting budgets for health services, examining how efficiently health services are being used, and to produce measures of clinical performance and quality of care adjusted for case mix.

### Conclusions

Inclusion of a diagnosis based patient morbidity measure into prescribing models can explain a large amount of variability at both patient and practice levels. The use of patient based case mix systems should be explored further when examining variation in prescribing patterns between practices in the UK, in particular for prescribing volume and for specific therapeutic prescribing categories. In the longer term, case mix systems may prove useful in fairer allocation of budgets and in the production of case mix adjusted measures of performance.

**What is already known on this topic**

Prescribing by UK doctors is under increased scrutiny, with pressure to prescribe cost effectively
Studies have not explained well the large difference in prescribing rates between practices

**What this study adds**

Inclusion of a diagnosis based patient morbidity measure in prescribing models can explain a large amount of variability, both between and within practices
Patient based case mix systems may help in the allocation of budgets

## Notes

Contributors: RZO and AM conceived the study and wrote the manuscript. RZO designed and supervised the statistical analysis and did some of the statistical analysis. CO’S constructed the groups on the basis of the adjusted clinical group, did the statistical analysis, and commented on the manuscript. AI extracted the data from the General Practice Research Database and responded to queries about the data. IP acted as an expert on the General Practice Research Database and commented on the manuscript. RZO and CO’S contributed equally to the paper. RZO, CO’S, and AM are guarantors.

Ethical approval: This study was approved by the scientific and ethical advisory committee, responsible for the Medicines and Healthcare products Regulatory Agency General Practice Research Database.

Provenance and peer review: Not commissioned; externally peer reviewed.

## Notes

**Cite this as:** *BMJ* 2008;337:a238
